# Risks Associated with Prescribing Zolpidem: A Case Report and Literature Review

**DOI:** 10.1192/j.eurpsy.2024.1178

**Published:** 2024-08-27

**Authors:** S. Jacob, A. Dore, O. Ali, H. Raai, L. Troneci

**Affiliations:** Psychiatry, St. Barnabas Hospital, Bronx, United States

## Abstract

**Introduction:**

Zolpidem is a nonbenzodiazepine, which acts as a sedative- hypnotic that binds to GABA (A) receptors at the same location as benzodiazepines and increases GABA effects in the central nervous system (Kovacic *et al.* Oxidative medicine and cellular longevity 2009, 2(1), 52–57). Literature shows that behavioral changes including amnesia, hallucinations, and other neurocognitive effects are some of the known side effects (Edinoff *et al*. Health psychology research 2021, 9(1), 24927). We present a case about Ms. A, a female in her sixties with a history of major depressive disorder with psychotic symptoms who was brought into the hospital by the EMS under police custody after stabbing her granddaughter with a knife. During the evaluation she was dissociating with impaired memory of the circumstances of her presentation. Collateral information about Ms. A revealed that she had no history of being violent, or any history of psychoactive substance use. Ms. A’s home psychiatric medications consisted of Sertraline 100mg, Bupropion 150 mg, Zolpidem 5mg.

**Objectives:**

To better understand the potential risks with prescribing zolpidem in patient with insomnia.

**Methods:**

In depth literature review about zolpidem. In addition, observation of Ms. A in the emergency with a full medical workup including but not limited to urine drug screen, brain imaging, lumbar puncture, etc.

**Results:**

Ms.A medical workup was positive for a urinalysis revealing asymptomatic bacteriuria and she was treated empirically with cefdinir. Her medication regimen consisted of Bupropion 150 mg and Sertraline 100m, both daily. Zolpidem was discontinued and changed to Clonazepam 0.5mg for insomnia. She was also started on Olanzapine 5mg in the AM and 10mg in the PM. Her mental status was noted to have improved after discontinuation of Zolpidem. Patient received one dose in the hospital but after two days since discontinuation her mental status improved. Upon literature review previous reports have been published citing cases of patients on Zolpidem physically acting out while sleeping in a parasomnia-like behavior, with no recollection of memories upon awakening. (Inagaki *et al.* Primary care companion to the Journal of clinical psychiatry 2010, 12(6)). There are case reports of Zolpidem associated homicide (Paradis *et al.*The primary care companion for CNS disorders 2012, 14(4).

**Conclusions:**

One limitation of our study is the patient was noted to have a sudden change in behavior with altered mental status which may be attributed to an underlying asymptomatic bacteriuria. It should be noted that this may have been an incidental finding. This does not exclude the possibility of Zolpidem as the primary cause of the change of her altered mental status or further exacerbating the change in her mental status. Though Zolpidem can be therapeutic and safe, we as clinicians have to be aware of the potential side effects of Zolpidem when prescribing medications.

**Disclosure of Interest:**

None Declared

